# DISCRIMINATION AND DEPRESSION IN MID- TO LATE LIFE: THE ROLE OF OPTIMISM

**DOI:** 10.1093/geroni/igz038.1154

**Published:** 2019-11-08

**Authors:** Elliottnell Perez, Joseph Dzierzewski, Scott Ravyts

**Affiliations:** Virginia Commonwealth University, Richmond, Virginia, United States

## Abstract

he positive association between discrimination and depression is well-supported throughout the literature. Previous evidence exploring potential mechanisms suggest discrimination is associated with depression via changes in social cognition. The goal of the current study was to investigate whether optimism explained the relationship between discrimination and depressive symptoms in mid-to-late life. Furthermore, the study assessed whether this mediated relationship was moderated by race or sex. This study included cross-sectional and longitudinal secondary data analysis of 2453 middle-aged and older adults (M age=68.30,SD=8.93) from the Midlife in the United States study. Discrimination was measured using an 11-item count of the number of discriminatory events experienced. Optimism was measured using the 6-item Life Orientation Test. Depressive symptoms were assessed using a 7-item count of the number of symptoms experienced. Optimism mediated the relationship between discrimination and depressive symptoms cross-sectionally, 95% CI:[.012, .034], and longitudinally, 95% CI:[.008, .024]. There was no evidence of moderated mediation; however, sex did moderate the direct relationship between discrimination and depressive symptoms cross-sectionally, b=.10, 95% CI:[.001, .194], and longitudinally, b=.03, 95% CI:[.01, .05]. The current study extends the literature by providing cross-sectional and longitudinal support for optimism as a mechanism through which discrimination leads to depressive symptoms in older adults. Evidence also suggests that women experience greater depressive symptoms than men in response to discrimination. Future research may wish to examine the developmental course of observed relationships and the impact of multiple marginalized identities on these relationships.

